# Development of a single-tube, low-cost, analytical process to extract, separate and determine efavirenz and rifampicin plasma concentrations in HIV/TB co-infected patients

**DOI:** 10.1186/1758-2652-13-S4-P149

**Published:** 2010-11-08

**Authors:** D Fox, R O’Connor, P Mallon, G McMahon

**Affiliations:** 1 Dublin City University, School of Chemical Sciences, Dublin, Ireland; 2 National Institute of Cellular Biotechnology, Dublin City University, Dublin, Ireland; 3 HIV Molecular Research Group, University College Dublin, School of Medicine and Medical Sciences, Dublin, Ireland

## Background

Tuberculosis (TB) complicating HIV-1 infection is a persistent significant clinical concern, particularly in resource limited settings. Treatment of HIV-infected patients with TB often comprises efavirenz-containing antiretroviral regimens particularly when on TB treatment containing rifampicin. Although rifampicin may reduce EFV concentrations, little is known of the effect of the HIV virus or efavirenz on the pharmacokinetics of rifampicin, particularly in resource limited settings where the burden of disease exists. We aimed to develop a low-cost, simple analytical method for the accurate determination of efavirenz and rifampicin plasma concentrations.

## Methods

Using high-performance liquid chromatography (HPLC) employing UV detection (1050 series quaternary pump, 1100 series autosampler, a diode array detector (DAD) and a 1200 series degasser), we developed and validated a single tube column-based assay for the detection of rifampicin and efavirenz. Data was acquired and analysed using Agilent Chemstation for LC 3D software.

## Results

Recovery for plasma samples spiked with the drugs were >90% for rifampicin and its metabolite deacetylrifampicin and >70% for efavirenz. Intra- and inter-assay precision relative standard deviation (RSD) values were <4% in all cases. The assay was validated on 300µl sample with a runtime of 10 minutes and both drugs measurable to concentrations of 100ng/mL.

## Discussion

This relatively easy, UV-based assay can accurately detect efavirenz and rifampicin concentrations within a clinically relevant concentration range using standard chromatography equipment, making it potentially applicable to resource limited settings.

